# Exploring barriers to and facilitators of the implementation of home rehabilitation care for older adults with disabilities using the Consolidated Framework for Implementation Research (CFIR)

**DOI:** 10.1186/s12877-023-03976-1

**Published:** 2023-05-13

**Authors:** Haixia Wang, Yanyan Zhang, Shouwei Yue

**Affiliations:** 1 School of Nursing and Rehabilitation, Cheeloo College of Medicine, Shandong University, Jinan, China; 2 University of Health and Rehabilitation Sciences, Qingdao, China; 3 Rehabilitation Center, Qilu Hospital of Shandong University, Jinan, China

**Keywords:** Barriers, Facilitators, Home rehabilitation care, Older adults, Qualitative study

## Abstract

**Background:**

With global aging on the rise, the number of older adults with disabilities was also increasing exponentially. There has been growing international interest in home rehabilitation care as a new method for older adults with disabilities.

**Method:**

The current study is a descriptive qualitative study. Guided by the Consolidated Framework for Implementation Research (CFIR), semistructured face-to-face interviews were performed to collect data. The interview data were analyzed using a qualitative content analysis method.

**Result:**

Sixteen nurses with different characteristics from 16 cities participated in the interviews. The findings highlighted 29 implementation determinants of home-based rehabilitation care for older adults with disabilities, including 16 barriers, and 13 facilitators. These influencing factors aligned with all four CFIR domains that were used to guide the analysis and 15 of the 26 CFIR constructs. More barriers were identified in the CFIR domain of characteristics of individuals, intervention characteristics, and the outer setting, while fewer barriers were identified in the inner setting.

**Conclusion:**

Nurses from the rehabilitation department reported many barriers related to the implementation of home rehabilitation care. They reported facilitators to the implementation of home rehabilitation care despite the barriers, which provided practical recommendations for directions to be explored by researchers in China and elsewhere.

**Supplementary Information:**

The online version contains supplementary material available at 10.1186/s12877-023-03976-1.

## Background

Populations are aging at a rapidly expanding rate worldwide due to people being healthier and living longer [1]. China has the largest population in the world, with over 260 million people aged over 60 years and 190 million people aged over 65 years in 2020. The aging process has led to older adults at risk of suffering from chronic and multiple age-associated diseases, which could lead to disabilities, especially physical disability and cognitive impairments [2]. The need for long-term care and rehabilitation provided by hospitals or families is often a consequence of disability, and this additional care can lead to higher medical costs [3]. Therefore, the shift from hospital to home care has been actively implemented in many high-income countries as a potentially more effective way to enhance the quality of life of older adults with disabilities [4, 5].

Home rehabilitation care is an important part of home health services, mainly for patients with disabilities to provide individual care services in the home environment [6]. Some governments have responded to the issues of an aging population to improve their outcomes and decrease costs by introducing home rehabilitation care for older adults with disabilities in their homes [7, 8]. However, the results of current studies on home rehabilitation care are still controversial [9, 10]. Some studies have shown that home-based rehabilitation care may be associated with improved quality of life and physical function compared to institutional care. However, home alternatives to institutional care may be associated with an increased risk of hospitalization [11]. The potential of home rehabilitation care to improve older adults with disabilities regain physical function is not clear, especially in China [12].

Home rehabilitation care is in the initial stage of development in China. The “Opinions on Accelerating the Development of Rehabilitation Medical Work” report released in 2021 emphasized that we must continue to create new types of rehabilitation services, and actively develop community and home rehabilitation services in China [13]. Home rehabilitation care for older adults with a disability is one important part of these services. However, there may be some obstacles to the implementation of home rehabilitation care in the Chinese cultural environment.

The Consolidated Framework for Implementation Research (CFIR) proposed by Damschroder et al. [14] is one of the most widely used theoretical frameworks. CFIR covers more than 500 papers from 13 disciplines and allows for a multidimensional and multilevel analysis of pre- and post-implementation influences. The CFIR was translated and introduced into China by Chinese scholars in 2021 [15]. Fewer studies have applied the CFIR to explore facilitators or barriers to the implementation of home rehabilitation care in China [16–18]. Thus, this study aimed to explore and describe barriers to and facilitators of the implementation of home care for older adults with disabilities in Shandong, China.

## Methods

### Study design

This study employed qualitative descriptive research, which proved to be the most effective method for providing a direct description of the phenomena [19]. The qualitative descriptive research approach was appropriate for the goal of this investigation. That is, we investigated rehabilitation nurses’ perspectives of barriers and facilitators to home rehabilitation care implementation. The study’s reporting followed the rules for consolidated criteria for reporting qualitative research (COREQ) [20].

### Participants and setting

All participants were nurses from various hospitals in Shandong Province who were conducting specialized training for rehabilitation care at Qilu Hospital’s rehabilitation department between June and July 2022. The Department of Rehabilitation of Qilu Hospital of Shandong University is a training center for rehabilitation specialists in Shandong Province. It is Shandong Province’s most well-known major tertiary center, providing high-quality rehabilitation treatment for older adults suffering from neurological and cerebrovascular illnesses. Purposive sampling was employed to assure the selection of individuals who might give greater insight into the phenomena of interest. Eligibility requirements for nurses included: (1) obtaining a nurse practitioner certificate; (2) obtaining a rehabilitation nursing specialist certificate; and (3) working in rehabilitation care for more than 3 years. Based on the above inclusion criteria we purposefully selected nurses who met the criteria as the interviewees for this study. In this study, the sample size was selected using the criterion of no duplication of information and saturation of the sample size [21].

### Data collection

The interview outline was first formed according to the CFIR guides. The CFIR is a meta-theoretical framework that consists of 39 constructs and five domains. The domain “process” refers to the implementation process and was not therefore used in this preimplementation phase of the study. Our collaborative team consisted of experts in the fields of rehabilitation, rehabilitation nursing, and implementation science. In addition, 3 nurses working in wards of Qilu Hospital of Shandong University were selected to conduct preinterviews according to the CFIR guidelines, and a primary interview guide was formed after the preliminary modification of the interview outline based on the results of the preinterview. The final version of the interview guide was developed after brainstorming with a team of experts.

Data collection was conducted during semistructured, face-to-face interviews [22]. All participants completed a simple information questionnaire ahead of time, and the interviews were held in a quiet, undisturbed room. We recorded the interviews using a tape recorder with the interviewee’s permission. Data collection and analysis were iterative procedures to allow new insights from the preliminary interviews to guide and enhance data collection for the follow-up interviews.

The first individual interview was conducted in Chinese, and the transcript was translated by a professional translator into English. The collaboration team had meetings to discuss the process of data collection and analysis, transcripts, and results. Then, the interviews were conducted in Chinese and translated concurrently by a professional translator during the interviews. Following the team discussions, the remaining interviews were completed in Chinese. Additional file 1 contains the interview guide.

### Qualitative analysis

Data were analyzed using the traditional content analysis method [23]. The reading of the raw data and familiarization with its content served as the foundation for the subsequent analysis of the data. To begin open coding, the researcher first examined the data at least twice to gain a sense of it as a whole. The second step was to highlight the key ideas and concepts. The third step was to categorize the related and comparable codes. Initially, the individual researchers (WH, ZY) coded the transcripts independently from each other. The results were compared and discussed in regular consensus meetings. By doing this, as well as a detailed documentation of the research process, both the criterion of intersubjective traceability and a significant quality criterion for qualitative research were fulfilled [24, 25]. Version 11 of NVivo was used to perform the data analysis.

### Ethical considerations

We obtained ethical approval from the institutional review board (2021-R-153) prior to the commencement of this study. All participants provided informed written consent. Voluntary participation and confidentiality of the data were ensured.

## Results

Finally, 16 participants from 16 hospitals participated in this study. Participants comprised 4 head nurse supervisors, 5 head nurses, and 7 nurses. All participants were female, and their ages ranged from 25 to 50 years. The majority of the participants had 2–31 years of work experience and care experience for older adults with disabilities. The length of the interviews ranged from 20 min to 55 min. Participant demographic characteristics are summarized in Table 1. The findings highlighted 29 implementation determinants of home-based rehabilitation care for older adults, including 16 barriers and 13 facilitators. These influencing factors aligned with all four CFIR domains that were used to guide the analysis and 15 of the 26 CFIR constructs (see Table 2). More barriers were identified than facilitators in the CFIR domain of characteristics of individuals and intervention characteristics, while fewer barriers were identified in the inner setting.

**Table 1:** Demographic characteristics of the participants (*N* = 16)

	Age	Hospital level	City	Title	Years of work	Education
1	40	Grade IIA	Zibo	Head nurse supervisor	19	College diploma
2	32	Grade IIC	Liaocheng	Head nurse	6	Bachelor’sdegree
3	32	Grade IIA	Weihai	Head nurse	13	Bachelor’sdegree
4	29	Grade IIB	Zaozhuang	Nurse	5	Bachelor’sdegree
5	29	Grade IIA	Weifang	Nurse	5	Bachelor’sdegree
6	50	Grade IIIA	Jinan	Head nurse supervisor	30	Bachelor’sdegree
7	45	Grade IIB	Linyi	Head nurse supervisor	24	Bachelor’sdegree
8	39	Grade IIA	Dezhou	Head nurse	12	College diploma
9	28	Grade IIIA	Qingdao	Nurse	5	Bachelor’sdegree
10	30	Grade IIA	Yantai	Nurse	5	Bachelor’sdegree
11	25	Grade IIB	Heze	Nurse	2	Bachelor’sdegree
12	29	Grade IIA	Jining	Nurse	5	Bachelor’sdegree
13	50	GradeIIIB	Dongying	Head nurse supervisor	31	Bachelor’sdegree
14	45	Grade IIB	Taian	Head nurse	23	Bachelor’sdegree
15	39	Grade IIA	Binzhou	Head nurse	12	College diploma
16	28	Grade IIIA	Rizhao	Nurse	5	Bachelor’sdegree

**Table 2:** Barriers and facilitators to implementation of home-based rehabilitation care

CFIR domains & constructs	Home-based rehabilitation care
**Characteristics of individuals**	**5 barriers, 1 facilitator**
Knowledge and Beliefs	2 barriers
Self-efficacy	1 barrier
Personal Attributes	2 barriers, 1facilitor
**Intervention characteristics**	**7 barriers, 4 facilitators**
Intervention Source	1 barrier 1 facilitator
Relative Advantage	2 facilitators
Adaptability	1 barrier
Trialability	1 facilitator
Complexity	3 barriers
Cost	2 barriers
**Outer Setting**	**2 barriers, 2 facilitators**
Patient Needs and Resources	1 facilitator
Cosmopolitanism	1 barrier
External Policies and Incentives	1 barrier, 1 facilitator
**Inner setting**	**2 barriers, 6 facilitators**
Structural Characteristics	2 barriers
Tension for Change	1 facilitator
Organizational Incentives and Rewards	3 facilitators
Available Resources	2 facilitators

### Characteristics of individuals

Five barriers and one facilitator were identified in three CFIR constructs for the characteristics of individuals. Participants reported a lack of knowledge and confidence in implementing home-based rehabilitation care. Most participants had never heard of home-based rehabilitation care for older adults with disabilities. Participants also reported concerns about implementing home-based rehabilitation care, especially if unexpected situations occurred. Participant 16 reported, *“What should a nurse do if an adverse event occurs while performing a relevant nursing operation? For example, if the patient’s condition suddenly deteriorated? This makes us nurses very unconfident when carrying out home rehabilitation care.”* Some nurses had low self-efficacy for implementing home rehabilitation care. Participant 9 stated, *“It is very difficult for me to carry out a new intervention; I lack confidence in myself and feel that I may not have the capacity to carry out home rehabilitation care.”*

Both barriers and facilitators were found in the individual constructs. Characteristics of nurses could affect the implementation of home rehabilitation care. Older age and poor communication were identified as barriers to the implementation of home rehabilitation care, while being responsible was identified as a factor that facilitated the implementation of home rehabilitation care. Participant 2 said, *“Older nurses are generally in a state of suboptimal health, and they may have difficulty physically handling the work of home rehabilitation care.”* Participant 8 reported, *“Some nurses are not good at communicating with patients and are introverted. These nurses are not suitable.”* Participant 16 stated, *“It is critical that nurses are responsible. The nurse goes to the patient’s home to provide relevant care, and the nurse must be responsible for the patient.”*

### Intervention characteristics

Seven barriers and four facilitators were identified in six of eight CFIR constructs of intervention characteristics. Nurses preferred that the evidence on home-based rehabilitation care be internally developed. Participant 4 said, *“the process of implementing home-based rehabilitation care varies greatly from region to region, especially in rural and urban areas. Internal development can be adapted accordingly.”* Some nurses also believed that the intervention program formulated at the national level is more authoritative. Participant 6 stated, *“More authoritative ah, programs, come from our national level. Otherwise, the program is only formulated by individual hospitals, and the implementation time may not be long.”*

The majority of participants believed that home rehabilitation care had a great advantage over ward care for older adults who were bedridden. With increasing aging, the demand for home rehabilitation care will increase for older adults with disability, and home rehabilitation care provides great convenience for older adults with disability. Participant 11 said, *“There is a great need for home rehabilitation care for older adults who are bedridden and in more stable conditions, which can significantly reduce medical costs.”* Participant 6 stated that *“home rehabilitation care is very convenient for patients, and patients do not have to go back and forth to the hospital.”* Participant 14 stated, *“Now that aging in China is more and more serious, our medical resources are limited; if the elderly can have home rehabilitation care, it is certainly good.”*

Interviewed nurses generally agreed that home-based rehabilitation care was triable. Participant 7 reported that *“home rehabilitation care is feasible to be piloted on a small scale in the community.”* Participant 13 said, *“I think it can be piloted on a small scale, such as changing urinary catheters and doing functional exercises for the limbs in the older adults.”* Participant 1 reported, *“We can choose some very basic nursing operations, such as changing the urinary catheter, intermittent catheterization, etc., first in the community near the hospital for a small pilot.”*

Although home rehabilitation care was considered to have relative advantages, the participants believed that home rehabilitation care has the characteristics of poor adaptability, high complexity, and high cost, which will hinder the implementation of home rehabilitation care to some extent. Adaptability refers to the degree to which home-based rehabilitation care can be adapted, tailored, and refined to meet local needs. The majority of participants felt that home rehabilitation care would not integrate well into their existing care. Participant 1 mentioned, *“Home rehabilitation care is in the patient’s home, but I work in a hospital, and it is difficult to adjust to the change in the workplace and the fact that I am already busy with my work.”* Participant 4 commented, *“Our daily ward work is very busy; there are many patients in the ward, and I have to be responsible for many patients. There is no way to combine that with home rehabilitation care.”*

Participants identified the enormous challenges of implementing a full range of home rehabilitation care. Safety was mentioned by all participants, and it was identified as the first important issue to consider. It is important and complex to ensure the safety of both the patient and the nurse. Participant 15 said, *“There are safety problems for patients…. we may do some invasive operations, such as…. the intubation of a urinary tube, and some adverse events may also occur.”* Participant 9 said, *“The first is safety…. if a nurse goes to the home alone…. especially if the older adults have a mental illness, the nurse might be attacked.”* Nurses also perceived the management of home rehabilitation care to be highly complex. Participant 8 said, *“The workplace is the patient’s home; the workplace is not fixed in a site, and the manager faces many challenges. For example, if a nurse-patient conflict occurs for some reason because it occurs in the patient’s home, the nurse’s personal safety may be at risk.”* In addition, Participant 5 said, *“Home rehabilitation care is a change in practice location from the existing ward-centered care; thus, a different type of nursing procedures is needed, which is another indication of the complexity of home rehabilitation care.”*

The cost of implementing home rehabilitation care is mainly labor and time. China is a large country with a large population, and the overall medical needs are enormous. The shortage of nurses is a constant problem, and the implementation of home rehabilitation care will require additional nursing workers to carry out this work, which greatly increases the demand for rehabilitation care workers. Participant 6 reported, *“We do not have enough nurses, and the work on the wards is very heavy, so where are the extra nurses who are going to do the home rehabilitation nursing work?”* Participant 11 said, *“The fundamental problem is that there are not enough nurses; there are not enough nurses in large hospitals and even fewer nurses at the primary level.”* Lack of time was also a common obstacle mentioned by nurses. Participant 4 stated, *“We need more time to do this work; we currently work both night and day shifts, and each nurse on the ward has to manage 6-8 patients, so what time do we have to do that? We need to rest, too; we are not robots.”*

### Outer setting

Three CFIR constructs of the outer setting included two barriers and two facilitators. Participants generally reported that home rehabilitation care takes full account of the health needs of older adults with disability, will greatly promote the development of home rehabilitation care. Participant 3 stated, *“I have had calls from patients’ carers who want me to come to their homes and change a urinary catheter for the patient.”* Participant 4 commented, *“My father was bedridden all the time and had to make an appointment to go to the hospital when he needed a urinary catheter change. The nurse came to his home to change his urinary catheter, which not only gave us great convenience but also saved on transportation costs.”*

Support from external policies was cited as an incentive to implement home-based rehabilitation care. Home-based rehabilitation care is part of a national strategy to make society more concerned about home-based rehabilitation. The participants were more than excited when they heard that home rehabilitation care has been proposed by the government. Participant 3 said, *“It is very good that the state is strongly advocating for it; it can only be implemented from top to bottom.”* However, a lack of support from local government policy was usually mentioned. For instance, Participant 7 stated, *“It is definitely good that the national government is proposing home rehabilitation care, but there is a lack of legislation or regulations and a lack of specific work processes from local governments.”*

A lack of cosmopolitanism is considered a major factor preventing the full advancement of home rehabilitation care. Hospitals are often not interlinked with other external organizations in China. Participant 6 mentioned that *“we work only in our own department, especially the nurses, and there are few opportunities to collaborate with other departments in our hospital and almost no collaborative interactions with other hospitals.”* Participant 12 reported that in all his years of practice, he had *“never encountered this situation and had not collaborated with other hospitals.”*

### Inner setting

Two barriers and six facilitators were identified in all four CFIR constructs of the inner setting. The age and immaturity of hospitals were considered to be critical barriers to the implementation of home rehabilitation care. Participant 5 suggested, *“Our hospital rehabilitation care is still in its infancy; ah, the department has not long been established and is understaffed.”* Participant 10 said, *“The number of nurses is small, and the nurses’ level of nursing operation skills is limited for a community clinic.”* However, there is great urgency to implement home rehabilitation care due to the problem of increasing aging. Participant 2 stated, *“I think it is very urgent; my grandfather is hemiplegic and bedridden. I feel it very much; there are a lot of people in my grandfather’s situation.”*

Participants felt that available resources, organizational incentives, and rewards are effective in facilitating the implementation of home rehabilitation care. Participants reported that professional training is essential for the implementation of home-based rehabilitation care, not only to improve the nurses’ professional skills but also to improve their communication skills and their ability to deal with emergencies. Participant 7 stated, *“I think specific training and preferably regular assessments are very important, so nurses have to meet the technical standards. The nurse’s communication skills are also very important.”* Participant 3 added, *“You do not know what’s going to happen suddenly in the patient’s home, and the ability of nurses to deal with the unexpected is also very important.”* On the other hand, nurses indicated that adequate funding is a prerequisite for the successful implementation of home rehabilitation care. For example, Participant 16 said, *“There must be sufficient funds, manpower, and financial resources – they are all needed. Some of the basic supplies for nursing care, rehabilitation equipment… without money it is definitely not possible.”* Participant 7 mentioned that such programs *“may increase the hospital’s burden because it needs more funds to cover the labor and materials costs.”* Participant 10 said, *“If the state is to cover this part of the funding, China’s aging problem is so serious that the fund must be very huge. In the long term, this approach may reduce the use of medical resources, but the initial financial investment must also be very huge.”*

Extrinsic incentives and rewards were considered to be strong facilitators for the successful implementation of home-based rehabilitation care. Participant 3 said, *“You know, for example, those nurses with families are still under some financial pressure, so they definitely want to earn more money to support their families; they would be happy to be given a salary increase if they were allowed to do this job and helped to promote the title.”* Participant 9 stated that *“as an adult, I still attach great importance to honor, awarding certificates and trophies.”*

## Discussion

This study conducted interviews with rehabilitation nurses with different characteristics from 16 cities in Shandong Province. The results of our study advance the understanding of the barriers and facilitators that provide more comprehensive rehabilitation care for older adults with disabilities and promote the development of home rehabilitation care in China.

Lack of knowledge and beliefs about home rehabilitation care in older adults with disabilities were identified as barriers in this study, which was in accordance with previous studies in the field of home care [26]. Of great concern was that a lack of knowledge and beliefs might lead to other barriers. This study found that participants lacked self-efficacy in the practice of home rehabilitation care, and they had little belief in their capability to provide home rehabilitation care to older adults with disabilities and address unexpected situations. In general, to address the barriers of self-efficacy, knowledge, and beliefs in personal characteristics, we should increase the promotion of home rehabilitation care. For example, short videos, brochures, and lectures can be used to increase nurses’ knowledge about home rehabilitation care and further encourage nurses to participate in home rehabilitation care [27]. In addition, it was found that the age and communication skills of nurses might influence the implementation of home rehabilitation care. Participants believed that older nurses are not suitable for home rehabilitation care. However, a study by Beatriz et al. [28] found that older patients prefer older nurses to provide rehabilitation care because they have more experience in care work. However, these inconsistent results were obtained from different perspectives of nurses or patients. Therefore, we should consider that nurses with experience and those of an appropriate age are more suitable for home rehabilitation care. Astrid et al. [29] found that nurses’ communication skills play an important role in home care, which is consistent with the results of our study. It was suggested that the personal characteristics of nurses should be considered when arranging home rehabilitation care to ensure the successful implementation of home rehabilitation care.

The construct of intervention characteristics had the highest number of factors perceived by participants, including seven barriers and four facilitators. Barriers were focused on the intervention source, adaptability, and complexity, as well as the cost of the characteristics of interventions. Home rehabilitation care is a new policy that has been introduced by the Chinese government in recent years. Participants confirmed that the new policy could ensure the implementation of home-based rehabilitation care. This finding is in accordance with the findings of previous studies [30]. However, some participants felt that only when the local government promulgates relevant policies and the hospital where they work develops a detailed implementation scheme could the implementation of home rehabilitation care actually be facilitated. Therefore, policy support from the Chinese government should come first, followed by local government policies in accordance with local conditions, to facilitate the implementation of home rehabilitation care. The complexity of home rehabilitation care was a serious concern for the participants, including safety, time, and management issues. Our research found that home rehabilitation care has safety issues for both patients and nurses, which is consistent with previous research results [31–33]. Recent research has shown that adverse events in home care are not rare. A study on home care safety from Canada reported that 5.5–15.1% of patients experienced at least one adverse event, such as falls or pressure-related ulcers, due to inadequate safety [34, 35]. Lack of time and workload were reported in the construction of costing. The barriers of lack of time identified in this study have also been commonly reported in many other studies in home care, as well as for other clinical issues. Nevertheless, this issue seems to be even more problematic for nurses in China. China faces a serious shortage of competent nurses who can address the health care needs of older people in an aging society. There were 4.7 million registered nurses in China at the end of 2020, and the ratio of registered nurses per 1000 people was 3.35 [36]. This is far below the average international ratio, which is 8.8:1000 [37]. Earlier studies in other countries also reported that insufficient staff leads to insufficient time to care for residents, which puts a great deal of pressure on staff and impedes their provision of home rehabilitation care [38]. Ma et al. argued that with the continuous development of network information science, we should make reasonable use of telemedicine information technology to improve the utilization of human resources for home rehabilitation care. Thus, the shortage of nurses should be alleviated as much as possible [39].

This study found that home rehabilitation care could greatly meet the health needs of older adults with disabilities at home in the construction of outer settings, supporting similar results in previous studies. The results of a meta-analysis showed that home rehabilitation care significantly improves health outcomes for older adults and significantly reduces health care costs [40]. In countries such as the United States, Canada, Australia, and Japan, home rehabilitation care is well established and developed [41, 42]. Although home rehabilitative care has been established in Canada, connections between tertiary hospitals and community services are also a common barrier [43]. In this study, all participants reported cosmopolitanism in different hospitals as a major barrier to implementing home rehabilitation care. Lack of collaboration represents the degree to which the hospital network and collaborate both internally and externally with other hospitals, which have been identified as inspiring teamwork and change [44]. We suggest that tertiary hospitals should improve the mechanism of sinking high-quality resources into home rehabilitation care, provide targeted support to secondary and lower hospitals, and improve the overall level of home rehabilitation care carried out by medical institutions. It is also necessary to develop interlinked medical systems so that collaboration between different regions and hospitals can be achieved more easily and quickly.

The construction of the inner setting had the highest number of facilitators perceived by participants at 6. These facilitators were focused on the tension for change, organizational incentives and rewards, and available resources. China is a country with a large population, and participants generally reported an urgent need for home rehabilitation care to improve the quality of life of older adults with disabilities as the aging process continues to increase [2]. In addition, home rehabilitation care requires nurses to have not only professional skills but also good communication skills and the ability to deal with emergency situations. Previous studies have noted that the professionalism of nurses is an important guarantee for home-based care [45]. The United States strictly requires that nurses working in home care should have at least 75 hours of training before they can start work [46]. It has also been found that nurses’ empathy, humor and emotional intelligence can greatly improve the quality of care provided at home [47]. Home rehabilitation nurses should be trained and rigorously and regularly assessed through a range of face-to-face, on-the-job and online methods, and schools should add courses on home care to ensure a higher quality home rehabilitation care workforce. However, participants commented that immaturity in the development of rehabilitation departments is considered to be a critical barrier to the implementation of home rehabilitation care, reflecting structural characteristics. In particular, the development of rehabilitation care in local primary hospitals is in its infancy. The development of rehabilitation nursing in China started relatively late, and most medical institutions in China are currently facing a shortage of rehabilitation nurses and poor nursing skills of rehabilitation nurses [48]. Therefore, it is especially important to strengthen the team of rehabilitation nurses and provide rehabilitation specialist training.

## Limitations

This study also has several limitations. It must be kept in mind that this was a small-scale study conducted in a small region in China, which in itself is of course a limitation. We included as many nurses as possible from different cities, hospital levels, titles and ages. However, this study did not include male staff, which might be a limitation in terms of the generalizability of the results. The researchers endeavored to be as responsive and rigorous as possible in regard to the informants’ narratives throughout the whole research process.

## Conclusion

Based on the CFIR theoretical framework, this study provides insight into the facilitators of and barriers to the implementation of home rehabilitation care, with the aim of providing a clear direction for the full implementation of home rehabilitation care. Home care managers should identify the factors that facilitate the implementation of home rehabilitation care and further eliminate the barriers that impede the implementation of home rehabilitation care to promote the successful implementation of home rehabilitation care and to improve the comprehensive quality of life of older adults with disabilities at home.

## Supplementary Information

**Additional file 1.** CFIR Guide.

## Data Availability

Transcripts from face-to-face interviews are among the study’s data. Due to the possibility of violating participant privacy, raw data cannot be made public. However, upon reasonable request, data are available from the corresponding author.
