# Supplementary material for: Exploring barriers to and facilitators of the implementation of home rehabilitation care for older adults with disabilities using the Consolidated Framework for Implementation Research (CFIR)
Source: BMC Geriatr. 2023 May 13;23:292. doi: 10.1186/s12877-023-03976-1 (PMC10183114; doi:10.1186/s12877-023-03976-1)
Supplement: Supplementary file 1 — Additional file 1. CFIR Guide. [file 12877_2023_3976_MOESM1_ESM.docx]

**CFIR Guide**

**Intervention Characteristics**

**Intervention Source**

1. Who developed the intervention?
   - What is your opinion of this group/individual?

**Evidence Strength & Quality**

1. What kind of information or evidence are you aware of that shows whether or not the intervention will work in your setting?
   - What evidence have you heard about from your own research? Practice guidelines? Published literature? Co-workers? Other settings?
   - How does this knowledge affect your perception of the intervention?
2. What kind of supporting evidence or proof is needed about the effectiveness of the intervention to get staff on board?
   - Co-workers? Administrative leaders?

**Relative Advantage**

1. How does the intervention compare to other similar existing programs in your setting?
   - What advantages does the intervention have compared to existing programs?

**Adaptability**

1. What kinds of changes or alterations do you think you will need to make to the intervention so it will work effectively in your setting?
   - Do you think you will be able to make these changes? Why or why not?
2. Who will decide (or what is the process for deciding) whether changes are needed to the intervention so that it works well in your setting?
   - How will you know if it is appropriate to make any changes?
3. Are there components that should not be altered?
   - Which ones should not be altered?

**Trialability**

1. Will the intervention be piloted prior to full-scale implementation?
   - [If Yes] Can you describe what your plans are for piloting the intervention?
   - [If Yes] What will the pilot look like?
2. Do you think it would be possible to pilot the intervention before making it available to everyone?
   - Why or why not?
   - Would this be helpful?

**Complexity**

1. How complicated is the intervention?
   - Please consider the following aspects of the intervention: duration, scope, intricacy and number of steps involved and whether the intervention reflects a clear departure from previous practices.

**Cost**

1. What costs will be incurred to implement the intervention?
2. What cost were considered when deciding to implement the intervention?

**Outer Setting**

**Patient Needs & Resources**

1. To what extent were the needs and preferences of the individuals served by your organization considered when deciding to implement the intervention?
   - Can you describe specific examples?
   - Will the intervention be altered to meet their needs and preferences?

**Cosmopolitanism**

1. To what extent does your organization encourage you to network with colleagues outside your own setting?
   - Are you able to attend local/national conferences? Other venues?

**External Policies & Incentives**

1. What kind of local, state, or national performance measures, policies, regulations, or guidelines influenced the decision to implement the intervention?
   - How will the intervention affect your organization's ability to meet these measures, policies, regulations, or guidelines?
2. What kind of financial or other incentives influenced the decision to implement the intervention?
   - How will the intervention affect your organization's ability to receive these incentives?
   - How will the new intervention affect payment or revenue for your organization?

**Inner Setting**

**Structural Characteristics**

1. How will the infrastructure of your organization (social architecture, age, maturity, size, or physical layout) affect the implementation of the intervention?
   - How will the infrastructure facilitate/hinder implementation of the intervention?
   - How will you work around structural challenges?
2. What kinds of infrastructure changes will be needed to accommodate the intervention?
   - Changes in scope of practice? Changes in formal policies? Changes in information systems or electronic records systems? Other?
   - What kind of approvals will be needed? Who will need to be involved?
   - Can you describe the process that will be needed to make these changes?

**Tension for Change**

- *Needs and Resources:*

1. Is there a strong need for this intervention?
   - Why or why not?
   - Do others see a need for the intervention?
2. How essential is this intervention to meet the needs of the individuals served by your organization or other organizational goals and objectives?
3. How do people feel about current programs/practices/process that are available related to the intervention?
   - To what extent do current programs fail to meet existing needs? Will the intervention meet these needs?
   - How will the intervention fill current gaps?

**Organizational Incentives & Rewards**

1. What kinds of incentives are there to help ensure that the implementation of the intervention is successful?
   - What is your motivation for wanting to help ensure the implementation is successful?
2. To what extent do you think your supervisor will consider your role in this implementation in your (next) evaluation? In his/her regard for your work or role?
3. Are there any special recognitions or rewards planned that are related to implementing the intervention?
   - Can you describe them?
   - Will these be targeted to groups/teams/units or individuals?

**Available Resources**

- *Resources may include operating and capital funding, dedicated personnel time (e.g. have new staff been hired, or is implementation a collateral duty), space, equipment, and/or information technology. Step through each to ensure all necessary resources are explored and fully described.*

1. Do you expect to have sufficient resources to implement and administer the intervention?
   - [If Yes] What resources are you counting on? Are there any other resources that you received, or would have liked to receive?
   - What resources will be easy to procure?
   - [If no] What resources will not be available?
2. How do you expect to procure necessary resources?
   - Who will be involved in helping you get what is needed?
   - What challenges do you expect to encounter?

**Characteristics of Individuals**

**Knowledge & Beliefs about the Intervention**

1. What do you know about the intervention or its implementation?
2. Do you think the intervention will be effective in your setting?
   - Why or why not?
3. How do you feel about the intervention being used in your setting?
   - How do you feel about the plan to implement the intervention in your setting?
   - Do you have any feelings of anticipation? Stress? Enthusiasm? Why?
4. At what stage of implementation is the intervention at in your organization?
   - How do you think the program is going?
   - Why do you say that?

**Self-efficacy**

1. How confident are you that you will be able to successfully implement the intervention?
   - What gives you that level of confidence (or lack of confidence)?
2. How confident do you think your colleagues feel about implementing the intervention?
   - What gives them that level of confidence (or lack of confidence)?

**Individual Stage of Change**

*1.*How prepared are you to use the intervention?

- - Knowledge stage (Precontemplation) - knowledge of key aspects of the intervention
  - Persuasion stage (Contemplation) - likes the intervention, discusses it with others, buys into it, has a positive view
  - Decision stage (Preparation) - intends to seek additional information and try it
  - Implementation stage (Action) - acquires additional information, uses intervention regularly, and has continued use
  - Confirmation stage (Maintenance) - recognizes benefits, has integrated the intervention into routines, promotes use to others

**Other Personal Attributes**

*1.What personal characteristics of nurses do you think affect how it is carried out?*
